# Primary culture of avian embryonic heart forming region cells to study the regulation of vertebrate early heart morphogenesis by vitamin A

**DOI:** 10.1186/1471-213X-14-10

**Published:** 2014-02-19

**Authors:** Inese Cakstina, Una Riekstina, Martins Boroduskis, Ilva Nakurte, Janis Ancans, Maija H Zile, Indrikis Muiznieks

**Affiliations:** 1 Laboratory of Biodosimetry and Bioanalytical Methods, Department of Biology, University of Latvia, Riga, Latvia; 2 Division of Pharmacy, Department of Medicine, University of Latvia, Riga, Latvia; 3 Division of Microbiology and Biotechnology, Department of Biology, University of Latvia, Riga, Latvia; 4 Department Food Science & Human Nutrition, Michigan State University, East Lansing, MI, USA

**Keywords:** Chicken heart forming region cells, *in vitro* culture, Retinoic acid, TGFβ2, Early cardiovascular development

## Abstract

**Background:**

Important knowledge about the role of vitamin A in vertebrate heart development has been obtained using the vitamin A-deficient avian *in ovo* model which enables the *in vivo* examination of very early stages of vertebrate heart morphogenesis. These studies have revealed the critical role of the vitamin A-active form, retinoic acid (RA) in the regulation of several developmental genes, including the important growth regulatory factor, transforming growth factor-beta2 (TGFβ2), involved in early events of heart morphogenesis. However, this *in ovo* model is not readily available for elucidating details of molecular mechanisms determining RA activity, thus limiting further examination of RA-regulated early heart morphogenesis. In order to obtain insights into RA-regulated gene expression during these early events, a reliable *in vitro* model is needed. Here we describe a cell culture that closely reproduces the *in ovo* observed regulatory effects of RA on TGFβ2 and on several developmental genes linked to TGFβ signaling during heart morphogenesis.

**Results:**

We have developed an avian heart forming region (HFR) cell based *in vitro* model that displays the characteristics associated with vertebrate early heart morphogenesis, i.e. the expression of Nkx2.5 and GATA4, the cardiogenesis genes, of vascular endothelial growth factor (VEGF-A), the vasculogenesis gene and of fibronectin (FN1), an essential component in building the heart, and the expression of the multifunctional genes TGFβ2 and neogenin (NEO). Importantly, we established that the HFR cell culture is a valid model to study RA-regulated molecular events during heart morphogenesis and that the expression of TGFβ2 as well as the expression of several TGFβ2-linked developmental genes is regulated by RA.

**Conclusions:**

Our findings reported here offer a biologically relevant experimental *in vitro* system for the elucidation of RA-regulated expression of TGFβ2 and other genes involved in vertebrate early cardiovascular morphogenesis.

## Background

Vitamin A and its active form, retinoic acid (RA) play an important role in cell proliferation, differentiation, morphogenesis and organogenesis during vertebrate development [1]. RA is involved in embryonic cell patterning, myogenic differentiation, chondrogenesis, central nervous system development and cardiovascular development [1-3]. RA regulates gene expression by binding specific nuclear transcription factors, retinoic-acid-receptors (RARs) and retinoid-X-receptors (RXRs), which interact with cognate promoter sequences, retinoic acid response elements (RAREs) [4,5] in target genes.

The cardiovascular system is the first functioning system to develop in the vertebrate embryo. At various stages during avian embryogenesis the defects in cardiovascular development can be caused both by a deficiency or an excess of vitamin A [6].

During a short time span during avian embryonic development, at the Hamburger and Hamilton stage 8 (HH 8) or at the 4–5 somite stage (ss), RA is indispensable for heart morphogenesis and for the survival of the avian embryo [3,6,7]. In the absence of vitamin A at this critical developmental window, morphogenesis of the posterior region of the primitive heart is impaired, the newly developing inflow tracts (IFTs) close inappropriately, and the sino-atrial tissue fails to differentiate [7]. Vitamin A-deficient (VAD) embryos die by day four of development with gross abnormalities in the developing heart and central nervous system. The abnormal development can be prevented by the administration of all-trans-RA to the VAD embryo during or prior to the critical RA-requiring developmental window at the 4–5 ss of embryogenesis [7].

The transforming growth factor beta (TGFβ) superfamily is a large group of multifunctional, structurally related cytokines that are major regulators of normal growth and development in multi-cellular organisms [8,9]. Expression of TGFβs and their cell surface receptors is universal and widespread throughout development. TGFβ canonical signaling pathway involves the intracellular mediators SMADs [10,11], although alternative options, e.g. the MAPK/ERK pathway, have also been identified [12]. TGFβs are expressed in three isoforms, TGFβ1, TGFβ2 and TGFβ3, each having specific, non-overlapping roles in cellular functions [13]. TGFβ2 is most strongly linked to heart morphogenesis [14]. TGFβ2-null mice exhibit cardiac, lung, craniofacial, limb, eye, ear and urogenital developmental defects [15].

TGFβ2 is expressed early in chick embryogenesis. At the 3–4 ss, it is localized in the notochord, neural tube and splanchnic mesoderm, the developmental progenitor of the heart forming regions [16]. Later in embryogenesis, TGFβ2 is expressed in the heart inflow tracts during the early looping stages, in the atrio-ventricular canal and in the heart outflow tract [11]. TGFβ2 is required to secure epithelial-mesenchymal transition during the development of endocardial cushions [17].

Regulation of TGFβ signaling by RA is well documented [9,11,13,14]. Depending on the cell type, RA can either induce or repress TGFβ expression [18]. Recent *in ovo* studies with quail embryos demonstrated that RA signaling is required for the normal development of the avian heart IFTs and that TGFβ2 is involved in this RA-regulated event. This was evidenced by the observation that in the absence of RA TGFβ2 was over-expressed in all embryonic cell layers, suggesting that RA acts as a negative regulator of TGFβ2 synthesis during embryogenesis [14]. Similarly, elevated ectopic TGFβ2 protein accounts for the heart septation defects and abnormalities in the outflow tracts of RA receptor mutant mice [1,19]. Since the TGFβ2 gene does not have RA response elements [10], the mechanism of regulation of TGFβ2 by RA is expected to be indirect and warrants further investigation.

The aim of this research was to develop a reliable *in vitro* model for the study of RA-regulated TGFβ2 expression during early vertebrate heart morphogenesis. Studies of transgenic mice with knock-outs or mutations of the RARs have provided important information about the role of TGFβ2 in RA-regulated events during advanced stages of heart formation, but have not addressed the role of this important growth regulator in the critical early events of heart morphogenesis. Using an *in vitro* chicken embryo heart-forming region (HFR) cell culture model we provide new insights into the role of TGFβ2 during the initial molecular events of heart building.

In order to obtain a better understanding of TGFβ2 function during early cardiovascular development as well as to expand the application of the model, we examined several genes known to be RA-regulated during embryonic cardiovascular development and also linked to TGFβ2. Vascular endothelial growth factor (VEGF-A) was a gene of great interest, since *in vivo* work has shown that the formation of the endothelial linkage of the heart to the primordial vasculature is an integral part of the early heart building events and that this process is RA-regulated and indirectly linked to TGFβ2 [6,14]. Furthermore, embryonic vasculogenesis and angiogenesis are known to be RA-regulated and thus likely involve VEGF-A [20,21]. Another RA-regulated gene is fibronectin (FN) [21,22], an extracellular matrix component essential for many morphogenesis events and a prominent component of the interface between the endoderm and mesoderm of the HFR, thus involved in building the embryonic cardiovascular system. Additionally, it has been reported that FN is a target of TGFβ signaling pathway [23]. Similarly, neogenin (NEO) is known to be induced by TGFβ signaling [23]. This multifunctional developmental protein was initially studied in neurogenesis [24], but recent studies have shown it to be an important regulator of organogenesis in the embryo [24]. Furthermore, NEO is a receptor for the bone morphogenesis proteins (BMPs) [25]. Since BMP-2 is regulated by RA during *in ovo* heart morphogenesis [26], the above observations warranted an examination of potential links of NEO to the RA-regulated heart morphogenesis.

In this paper we describe the development of a chicken embryo HFR cell culture to obtain insights into RA-regulated TGFβ2 signaling during the initial stages of heart morphogenesis under conditions where the concentration of RA is easily modified and RA is the only modulator during the growth of the culture. The model will allow the elucidation of the molecular mechanisms of signal transduction by TGFβ2 as well as of other important genes involved in RA-regulated early heart morphogenesis.

## Results

### Development of chicken HFR explant and cell culture model

**Figure 1:**
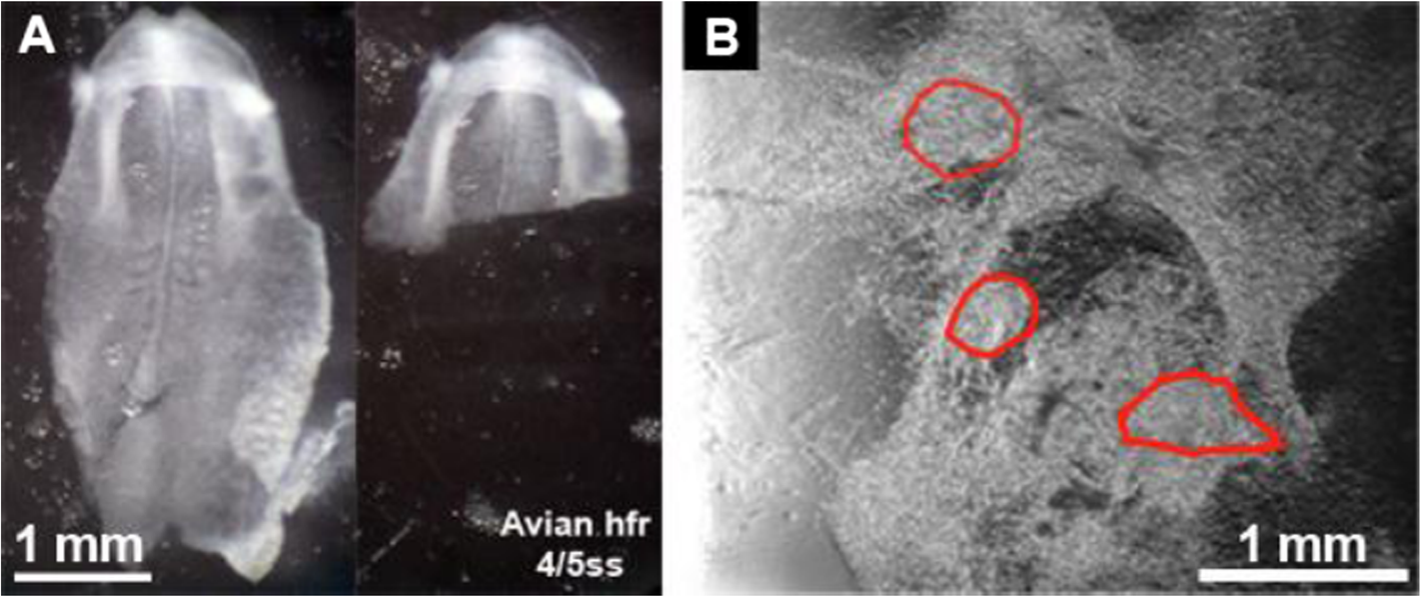
**Development of chicken embryo HFR explant in culture. (A,** left**)** Chicken embryo at 4 ss; (**A**, right); dissected heart forming region explant from 4–5 ss embryo; **(B)** 4–5 ss heart forming region explant after one day of cultivation in Endo-Grow medium. Red lines mark the borders of contractile tissue developed within a day in culture (see Additional file 1). Contractile tissues were observed by microscopy and documented using video camera. hfr, heart forming region; ss, somite stage. Scale bars, 1 mm.

Explants of the HFR (Figure 1A) tissue from 1–10 ss embryos were obtained after 36 to 42 h incubation of freshly fertilized eggs at 37°C and placed in separate wells on 24-well plates. DMEM/10% FBS medium was used to propagate all types of adherent cells; Endo-Grow medium was used to promote growth of endothelial lineage cells. Contractile cardiomyocytes (Figure 1B, Additional file 1) were observed after 24 h of cultivation in both media, although the development of contractile tissue in DMEM medium was less efficient in comparison to that in Endo-Grow.

After one day of incubation, the formation of contractile tissue from the 1–2 ss explants was greater in the Endo-Grow medium as compared to that grown in DMEM medium (Table 1). The greatest amounts of contractile cardiomyocytes were formed when explants from 3–5 ss embryos were cultured in Endo-Grow medium. The capacity of explants to develop contractile tissue diminished significantly using later stage embryos.

**Table 1:** Numbers of chicken HFR explants forming contractile tissue aggregates

	** Developmental stage**		
Media	1–2 ss	3–5 ss	7–10ss
DMEM	12%*, (1 of 8)	14%, (1 of 7)	0%, (0 of 4)
Endo-Grow	37%, (3 of 8)	78%, (7 of 9)	25%, (1 of 4)

The total number of explants placed in culture media is shown in parentheses. *Percentage of explants forming contractile tissue aggregates within 24 h of cultivation. Experimental details are described in Methods.

**Figure 2:**
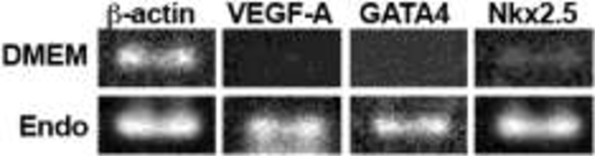
**Expression of heart development marker genes in chicken heart forming cell culture.** Primary heart forming region (HFR) cells from 3–5 ss chicken embryos were cultured in DMEM and Endo-Grow medium and analyzed for VEGF-A, GATA4 and Nkx2.5 mRNA after 3 d in culture. 30 cycles of PCR amplification were run with cDNA isolated from 150,000 HFR cells. β-actin was used as a reference.

In RT-PCR assay, HFR cells from explants (n = 10–15) of 3–5 ss embryos were grown in Endo-Grow media and gave strong intensity bands for the mRNAs of the VEGF-A, cardiac transcription factors GATA4 and Nkx2.5 genes, all known to be involved in cardiovascular morphogenesis. HFR cells grown in DMEM produced weak bands for VEGF-A and Nkx2.5 and no bands for GATA4 transcripts (Figure 2).

Homeobox gene (HoxB1) is involved in early development of vertebrate embryos and is regulated by RA both *in vitro*[27] and *in vivo*[28]. HoxB1 has RA response elements within its promoter sequence and thus is a direct RA target gene [27].

**Figure 3:**
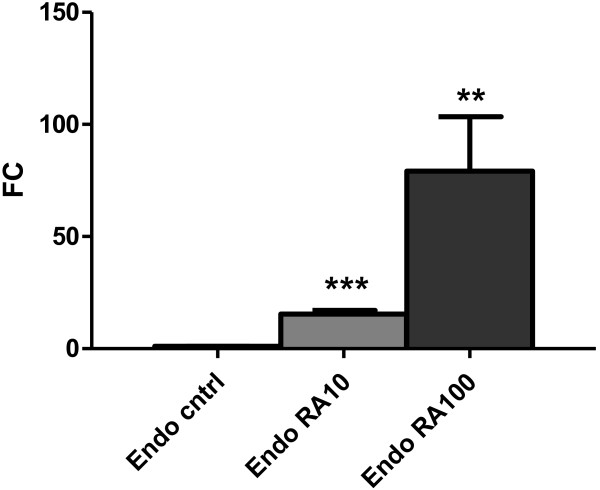
**Expression of HoxB1 in chicken heart forming cell culture is responsive to presence of retinoic acid (RA) activity.** Primary heart forming region cells from 2–6ss chicken embryos were cultured in Endo-Grow media in presence of RA. Relative quantification of HoxB1 mRNA in comparison to control cells with no added RA. Standard deviations are indicated by error bars. The differences in comparison to the control at significance level of p < 0.005 are marked by (**); at p < 0.0001 by (***).

When RA was added to the medium, HoxB1 gene mRNA expression increased 15 times and 78 times in Endo-Grow media supplemented with 10 nM and 100 nM RA, respectively (Figure 3).

### Effect of exogenous RA on TGFβ2 gene and protein expression in cultured HFR cells

Chicken HFR cells were grown in DMEM and in Endo-Grow media supplemented with exogenous RA at a concentration of 10 nM (which approximates the concentration of RA in the early avian embryo [29], or at the excess level of 100 nM. Cell growth medium was collected after 3 d of cultivation and used for TGFβ2 ELISA assay on the same day.

**Figure 4:**
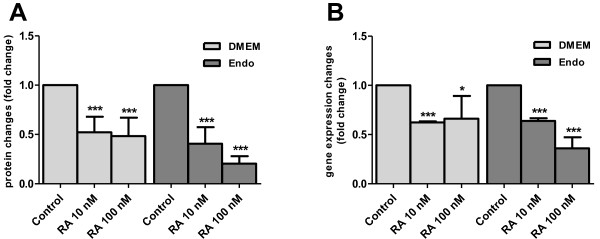
**Retinoic acid regulates TGFβ2 in chicken heart forming cell culture.** Primary heart forming region cells from 3–5 ss chicken embryos were grown in culture for 3 d in either DMEM or Endo-Grow (Endo) media in the presence or absence of added retinoic acid (RA). **(A)** Relative quantification of TGFβ2 total protein in the cultivation media in comparison to control cells with no added RA. **(B)** Relative quantification of TGFβ2 mRNA in comparison to control cells with no added RA. Standard deviations are indicated by error bars. The differences in comparison to the control at significance level p < 0.05 are marked by (*), at p < 0.005 by (**); at p < 0.0001 by (***).

Figure 4 shows that the accumulation of total TGFβ2 protein and mRNA was suppressed at both 10 nM and 100 nM RA concentrations in comparison to controls without added RA. When RA was added, TGFβ2 protein decreased by 50% in the cultures grown in DMEM and by 60% and 80% when grown in Endo-Grow medium (Figure 4A). TGFβ2 mRNA synthesis was also decreased in the presence of exogenous RA. In DMEM media the accumulation of TGFβ2 transcripts was reduced by approximately 35% with both RA concentrations; in Endo-Grow media TGFβ2 transcripts were reduced by 40% at 10 nM RA and by 60% at 100 nM RA in comparison to controls without added RA (Figure 4B).

### Effect of exogenous RA on the expression of TGFβ/SMAD regulated genes and on VEGF-A mRNA synthesis

**Figure 5:**
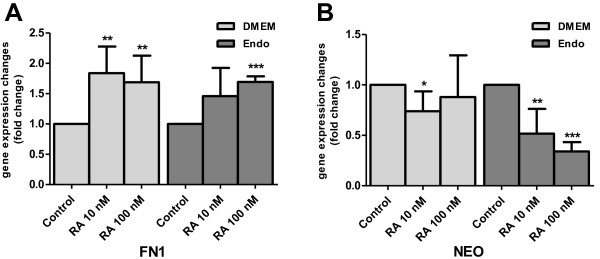
**Retinoic acid regulates the expression of FN1 and NEO in chicken heart forming cell culture.** Primary heart forming region cells from 3–5 ss chick embryos were cultured for 3 d in either DMEM or Endo-Grow (Endo) media in the presence or absence of retinoic acid (RA). **(A)** Relative quantification of FN1 mRNA in comparison to FN1 mRNA in control cells with no added RA. **(B)** Relative quantification of NEO mRNA in comparison to NEO mRNA in control cells with no added RA. Standard deviations are indicated by error bars. The differences in comparison to the control at significance level p < 0.05 are marked by (*); at p < 0.005, by (**); at p < 0.0001, by (***).

The expression of FN1, NEO and VEGF-A genes characterize the development of extracellular matrix, neuronal tube and vascular endothelium, respectively, within the embryonic tissue. All these processes contribute to heart morphogenesis. The expression of FN1 and NEO genes is known to be up-regulated by TGFβ through the SMAD signaling pathway in human fibroblast culture [23]. If in the chicken primary HFR cell cultures these genes were to function as in the human fibroblasts, then the addition of RA, known to down-regulate TGFβ2 in the avian embryo [14], would also cause a decrease in FN and NEO expression. However, there was a statistically significant increase in FN1 mRNA in HFR cells when cultivated in either DMEM or Endo-Grow medium in the presence of exogenous RA (Figure 5A). In contrast, NEO mRNA expression in HFR cells decreased in the presence of exogenous RA (Figure 5B), paralleling the down-regulation of TGFβ2 expression observed earlier (Figure 4). This response was most pronounced in cells cultured in Endo-Grow medium, where NEO mRNA concentration decreased to 51% and 34% of the mRNA levels observed in controls cultured without RA supplementation. NEO gene expression in cells grown in DMEM in the presence of exogenous RA resulted in a slight decrease at 10 nM RA, while the decrease at 100 nM RA was not statistically significant.

**Figure 6:**
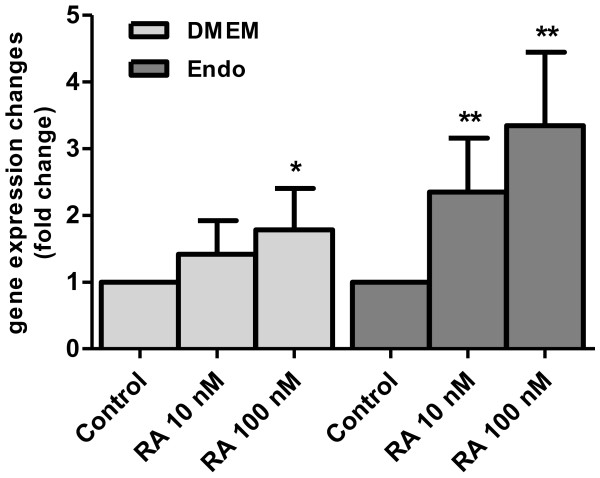
**Retinoic acid regulates the expression of VEGF-A in chicken heart forming cell culture.** Primary heart forming region cells from 3–5 ss chicken embryos were cultured for 3 d in either DMEM or Endo Grow (Endo) media in the presence or absence of added retinoic acid (RA). Relative quantification of VEGF-A mRNA in comparison to VEGF-A mRNA in control cells with no added RA. Standard deviations are indicated by error bars. Differences in comparison to the controls at significance level p < 0.05 are marked by (*); at p < 0.005, by (**).

VEGF-A mRNA expression in the HFR cells cultured in DMEM with 10 nM RA or 100 nM RA was increased by 42% and 78%, respectively in comparison to controls without supplemented RA. The effect was even more pronounced when the cells were cultured in Endo-Grow medium, as there was a 135% increase in the VEGF-A mRNA expression with 10 nM RA and a 235% increase in the presence of 100 nM RA (Figure 6).

## Discussion

There have been many studies on the role of retinoids in vertebrate heart development [30], however only a few have addressed early heart morphogenesis during physiologically relevant conditions [3,6]. The present study for the first time offers a biologically significant and valid *in vitro* primary cell culture model from early stage chicken heart forming regions (HFRs) that can be used to study vertebrate molecular mechanisms regulated by RA under conditions that closely mimic early events of vertebrate *in vivo* heart morphogenesis. The model is based on the published *in ovo* observations that in the avian embryo the requirement for RA begins at the 4–5 somite stage (ss) of neurulation [31,32]; this is also the time when heart morphogenesis is initiated and when the presence of RA is absolutely essential for normal embryonic development to take place [7].

Since vertebrate early heart development is comparable across species, the avian embryo offers an ideal model to study this most important early developmental event. To offer an alternative to the *in vivo* model for studies of retinoid function during early stages of heart morphogenesis, our strategy was to utilize the knowledge obtained from the *in ovo* studies to produce an *in vitro* model that could mimic the *in ovo* model system. We accomplished this goal by isolating HFR cells from 3–5 ss normal chicken embryos and culturing them under conditions that generate tissue characteristics and biological responses in the presence of retinoic acid. It is important to note that for the *in vitro* system described here it is critical to harvest the HFR tissue during the narrow 3–5 ss developmental window, as pointed out above.

We established that the cultured cells exhibited the expected characteristics of heart tissue, i.e. forming contractile tissue after 24 hr of growth in culture (Table 1, Figure 1B, Additional file 1), expressing the cardiogenic gene Nkx2.5, the cardiogenic transforming factor GATA4 and the vascular endothelial growth factor VEGF-A (Figure 2). Next, it was important to verify the responsiveness of the cultured cells to the vitamin A-active form, retinoic acid (RA). Although the Endo-Grow medium that was chosen for culturing the cells contained 0.1 nM RA, this level is significantly lower than the physiological level of RA reported for the normal early avian embryo, i.e. 5 nM [29], thus the expression of some RA-regulated genes may be weak in these cultures; the addition of 10 nM RA would be close to the physiological level of the early avian embryonic cells.

To ascertain that the chicken HFR cell culture is indeed a valid *in vitro* model for the elucidation of RA-regulated molecular events during early vertebrate embryogenesis, we modulated the expression of HoxB1, a direct RA target gene [27], in response to the presence of RA in the culture media (Figure 3) and verified that the cell culture represents a reliable system for the proposed studies.

Having established a viable and valid *in vitro* heart forming cell culture model for studying RA-regulated heart morphogenesis, we next examined in this model the expression of some of the genes that had been reported to be regulated by RA during early *in ovo* heart morphogenesis [6].

We focused on TGFβ2 because it is intricately involved in RA-regulated early heart morphogenesis and shares many global regulatory characteristics with RA [6]. Furthermore, endogenous RA is a critical negative *in vivo* regulator of TGFβ2 during avian embryogenesis. TGFβ2 mRNA and protein concentrations are increased in the vitamin A-deficient (VAD) quail embryo, and supplying a physiological amount of RA to the VAD embryo decreases and thus normalizes the expression of TGFβ2 [14]. In line with the reported *in ovo* observations [14], the presence of 10 or 100 nM RA in HFR cell culture caused a decrease in TGFβ2 mRNA and protein concentrations in the cultured cells (Figure 4). This finding provides evidence that the chicken HFR cell culture is a valid biological model to further examine the interrelationship between RA and TGFβ2 during early vertebrate heart morphogenesis.

Since TGFβ2 gene promoter region does not have RA response elements [10] which are required for a direct regulation by RA via its RAR/RXR complexes, an indirect mechanism must be involved. The expression of TGFβ2 is governed by two AP-1, two AP-2, two Sp1, and four CRE binding sites located in the promoter of this gene [33]. An indirect regulation of TGFβ2 by RA might be achieved by RA affecting one or more of these binding sites. It is known that the RA/RAR/RXR complex can bind to AP-1 blocking its activity to induce TGFβ2 expression [34]; discussed in [6]. Recent studies of amniotic cell membrane explants show that the RA/RAR/RXR complex can also interact physically with the transcription factor Sp1 preventing it from binding to its site in the TGFβ2 promoter [35]. Clearly, further analysis of the interactions of TGFβ2 promoter elements and DNA binding proteins *in vitro* are needed to elucidate the molecular mechanism of RA-regulated TGFβ2 expression during embryogenesis.

In preliminary studies with adult chicken heart cells and human heart cells isolated from the heart biopsies, we did not observe any effect on TGFβ2 gene expression when the RA concentration in the media was either increased or decreased (data not shown). This is likely due to differences in RA/TGFβ2 regulatory interactions in embryonic and adult cells, reflecting epigenetic changes during development. DNA methylation at CpG sequences may differentially affect the binding of the transcription factors involved in the regulation of TGFβ2 expression, e.g. AP-2 binding might be blocked [36], AP-1 binding might be stimulated [37], and CRE binding may be blocked or modified [38]. These observations emphasize the need for adequate model systems to study TGFβ2 expression at specific stages of development.

Studies with quail embryos have shown that when the early embryo is retinoid deficient, TGFβ2 is overexpressed in all cell layers in the anterior part of the embryo [14]. Thus, these ectopically elevated TGFβ2 levels could adversely affect many of its target genes. It was therefore important to examine RA-regulated developmental genes that have also been linked to TGFβ in other cells. One of the genes examined was FN, regulated both by RA [21,22] and TGFβ/SMAD [23]. Another gene of interest was NEO, a multifunctional developmental receptor linked to embryonic organogenesis [24]. In human fibroblast cell culture FN1 and NEO are regulated by TGFβ through the SMAD signaling pathway [23,39]. It was therefore of interest to examine the expression of these genes in our cell culture as it might provide insights into the role of TGFβ2 in the RA-regulated early heart morphogenesis. Since a GenBank sequence analysis of FN and NEO genes did not reveal the presence of RARE elements in their promoters, they are not direct RA target genes.

We found that in our chicken HFR cell cultures the baseline level of FN1gene expression was increased when RA was added to the cultures in both DMEM and Endo-Grow media (Figure 5A), however, it was not a dose-dependent increase. An explanation for this observation might be that FN1 is relatively insensitive to small changes of RA concentrations in the environment at this time point in development, and that higher levels of RA are required to affect its expression; the mechanism of this is not known. In contrast, FN1 was reported to be down-regulated by RA in mouse embryonic fibroblast cells NIH-3 T3 [40]. Further studies are needed both *in vivo* and in a culture model to clarify the RA/FN interactions during heart formation. It is, however, very likely that the FN1 expression observed in our HFR cultures is of biological significance since the expression of various other genes examined in this *in vitro* system was similar to that observed *in vivo*.

The expression of NEO gene in the cultured chicken HFR cells would suggest the presence of neuronal cell subpopulation that might have been derived from the early neuronal tube region located underneath the HFR of the embryo and that had not been completely removed during the preparation of the explants. However, since NEO regulates diverse developmental events [24], it may also be involved in cardiovascular development. Support for this hypothesis comes from our observation that in the cultured chicken HFR cells NEO is regulated by RA (Figure 5B) and this regulation appears to be via TGFβ2 signaling, since NEO expression in response to RA concentration in the Endo-Grow media paralleled that of TGFβ2 expression (Figures 4 and 5). Thus, our study is the first to report the novel observation that RA regulates NEO expression in the vertebrate embryo and at a critical time of heart morphogenesis. Since GenBank sequence analysis of this gene did not reveal the presence of RARE elements in its promoters, we hypothesize that in the cultured chicken HFR cells NEO is regulated by RA indirectly through a TGFβ2 signaling pathway. Although in most systems TGFβ signals are linked to SMAD transcriptional activity events [41], *in ovo* studies with quail embryos suggest that the RA-regulated TGFβ2 signaling during early heart morphogenesis is linked to SMAD- independent pathways [14]. Further studies are needed to determine if the RA-regulated NEO expression observed in the cultured chicken HFR cells is linked to heart morphogenesis and to TGFβ2 signaling, and if SMAD transcription factors are involved.

Studies with quail embryos *in ovo* have also demonstrated vitamin A requirement for embryonic vasculogenesis and for the generation of endothelial cell progenitors during early heart morphogenesis, suggesting that an insufficiency of endothelial cells may be a crucial factor in the VAD-related heart malformations [7,20]. Importantly, these *in vivo* studies have also revealed that vitamin A is required to build the link between the primordial heart and its blood supply, i.e. the cardiac inflow tract [6]. This event most likely involves VEGF-A which is required for angiogenesis and the organization of blood vessels [42]. RA-regulated VEGF-A expression involving TGFβ signaling has been reported in human embryonic stem cells [43]. The above observations prompted us to determine if VEGF-A is expressed in the cultured chicken HFR cells and if it is regulated by RA. Indeed, in these cultures we observed VEGF-A expression (Figure 2), which was significantly enhanced by added RA (Figure 6). It is very likely that the *in vitro* observed VEGF-A expression represents the *in ovo* situation. Sequence analysis of the VEGF-A promoter revealed two typical RARE sequences therefore RA might directly modulate VEGF-A mRNA synthesis. VEGF upregulation by RA has also been observed in smooth muscle [44] and in adipocytes [45], while Balmer and Blomhoff reported inhibition of VEGF by RA [46]. Additionally, studies with the synthetic derivative CD2409 showed that RA downregulates VEGF-A expression through activation of AP1 [47]. These contradictory observations could be explained by species differences and different cell types used, e.g. human keratinocytes [48], and tumor endothelial cells [49]. Furthermore, none of these publications specify which VEGF transcript variant was used in the studies.

Altogether, the cultured chicken HFR cell model is a biologically relevant system to examine RA-regulated vertebrate early heart morphogenesis under conditions that allow the manipulation of RA activity. Experiments on RA-regulated genes described here set the basis for future studies to elucidate the molecular mechanisms involved in vertebrate cardiovascular morphogenesis.

## Conclusions

In vertebrate developmental research, relevant *in vivo* models are not always accessible and in many instances *in vitro* models can provide alternative approaches to advance the field. We have developed a biologically relevant and valid *in vitro* primary cell culture model from early stage chicken heart forming regions that provides an excellent research tool to study retinoic acid (RA) regulated molecular mechanisms during vertebrate early heart morphogenesis under conditions that closely mimic *in vivo* heart morphogenesis. We have verified the usefulness of the *in vitro* model by confirming published *in vivo* observations regarding the regulation of TGFβ2 by RA during early heart formation, as well as by identifying additional RA-regulated genes, i.e. FN, NEO and VEGF-A that might be linked to early heart morphogenesis. The *in vitro* model described here will be a valuable addition to the experimental tools for vertebrate developmental research.

## Methods

### Avian heart forming region explant and cell cultures

Freshly fertilized Ross308 chicken eggs (poultry farm Kekava, Latvia) were incubated for 36–50 h at 37°C and used to obtain early embryonic tissue. The experiments with the early chick embryos followed the EU directive 2010/63/EU, Chapter I, Article 1, Paragraphs 3 and 4, as pertaining to the very early stages of vertebrate development. Embryos were dissected under microscope in cold phosphate buffered saline (PBS) and staged by their somite stage (ss) of development. Heart forming regions (HFRs) from chicken embryos at 1–2 ss, 3–5 ss and 7–10 ss were separated from the rest of the embryonic tissue, rinsed shortly in cold PBS, then transferred to cold, sterile DMEM (Dulbecco’s Modified Eagle Medium, containing no FBS), containing 1% penicillin/streptomycin and 2% fungizone (all from Invitrogen) to ensure a sterile environment during dissections. After dissections, explants were washed with sterile PBS, placed in either DMEM or Endo-Grow media (see below) and incubated in 24-well plates, one explant per well for 3 days at 37°C and at 5% CO2.

Dissected explants were also used to obtain cells for cultivation studies. For primary cell culture studies only explants from the 3–5 ss embryos were used, as this stage of avian embryonic development is the critical stage when heart morphogenesis is initiated and when the presence of RA is essential. This was corroborated in preliminary studies with cultured HFR explants from the 3–5 ss embryos as these explants formed a very high percentage of contractile heart tissue (Table 1). Primary cultures of 3–5 ss HFR cells were prepared from combined 10–15 explants collected in DMEM containing 1% penicillin/streptomycin and 2% fungizone, then trypsinized, centrifuged, the pellet suspended in either DMEM or Endo-Grow media (see below) and the cell suspension plated. One explant yielded approximately 5 × 10^4^ cells. Cells were counted and plated on 24-well plates with 2 × 10^4^ – 3 × 10^4^ cells/well. Explanted cells developed adherent monolayer within 3 d in culture.

The following two media were used in all cultivation studies: standard DMEM (Dulbecco’s Modified Eagle Medium) base, supplemented with 10% Fetal Bovine Serum (FBS) and 1% penicillin/streptomycin (all from Invitrogen), and endothelial cell growth media base, Endo-Grow (Millipore/Chemicon), supplemented with 2% FBS, 5 ng/ml Endothelial Growth Factor, 5 ng/ml basic Fibroblast Growth Factor, 15 ng/ml Insulin-like Growth Factor, 50 μg/ml ascorbic acid, 1 μg/ml hydrocortisone hemisuccinate, 0.75 U/ml heparin sulfate, 10 mM L-glutamine (all from Millipore/Chemicon) and 1% penicillin/streptomycin, according to the manufacturer’s protocol.

In studies with RA, both media were supplemented with all-trans-RA (Sigma) to a final concentration of 10 nM (3 ng/ml) or 100 nM (30 ng/ml). The HFR cell cultivation experiments in RA-supplemented media were replicated 5 times. Control cells were grown in media without added RA. All manipulations with RA were made in dim light.

#### ***Analysis of secreted TGFβ2***

The amount of total secreted TGFβ2 protein in medium was quantified by TGFβ2 DuoSet ELISA (R&D Systems, Cat.nr. DY302; the antibodies react with avian proteins). In brief, cell culture supernatants were collected and immediately used for ELISA. Supernatants were incubated with 1 N HCl at RT for 10 min to activate latent TGFβ2 and neutralized with 1.2 N NaOH in 0.5 M HEPES, according to manufacturer’s instructions. Assays were repeated 5 times with triplicate samples. The changes of secreted TGFβ2 in RA supplemented media were expressed as fold change of secreted TGFβ2 from control (media with no RA supplement). Statistical analysis was performed using GraphPadPrism v 5.0 software.

#### ***RT-PCR and real-time PCR analysis***

RNA from chicken HFR cell cultures was extracted using TRIzol reagent (Invitrogen) according to the manufacturer’s recommendations. The purity was assessed using Nanodrop ND-1000 and integrity was assessed by agarose gel electrophoresis. First strand cDNA synthesis was performed on total RNA using RevertAid™ M-MuLV Reverse Transcriptase (Fermentas). The quality of cDNA was tested in PCR reaction (25 cycles) with chicken housekeeping gene β-actin primers. Chicken VEGF-A, GATA4, Nkx2.5 and HoxB1 gene mRNAs were tested in obtained cDNA by standard PCR reaction (Veriti, Applied Biosystems), as follows: initial activation at 95°C for 5 min, followed by 30 to 35 cycles of melting at 95°C for 15 sec, annealing at 58 to 60°C for 20 sec and elongation at 72°C for 20 sec. All primer sequences are listed in Table 2.

**Table 2:** PCR oligonucleotide sequences used in this study

**Gene**	**Primer sequence**	**Product size (bp)**	**GeneBank**
chicken-β-actin	ctgaaccccaaagccaacag, ccagatccagacggaggatg	214	X00182.1
chicken VEGF	gcagagcgcggagttgtc, gtccaccagggtctcaattgtc	127	NM_001110355
chicken GATA4	cggcctctcttgtgccaact, gatggacctgctggcgtctt	203	U11887.1
chicken Nkx2.5	accccgcgtcctcttttctc, cccaccatctccagggtctg	197	NM_205164.1
chicken TGF-β2	cagtgggaagaccccacatc, tgaatccatttccagccaag	185	X59080.1
chicken FN1	cgcccctaccactctgacac, cagctctgcaacgtcctcct	175	XM_421868.2
chicken NEO	tccggatagctgccatgact, tggagtccagctcaccacaa	161	U07644.1
chicken HoxB1	aacctctcgccttccctaaa, agctgcttggtggtgaagtt	191	NM_001080859

Gene expression levels were quantified by real-time PCR on ABI Prism 7300 (Applied Biosystems) using Maxima SYBR Green/ROX qPCR Master mix (Fermentas). In brief, the reaction mixture in 20 μl total volume contained 15 ng single stranded cDNA, gene-specific forward and reverse primers (0.4 μM final concentration), 10 μl SYBR Green Master mix. PCR conditions were as follows: initial activation step at 95°C for 4 min, 40 cycles each of melting at 95°C for 15 sec and annealing/extension at 60°C for 40 sec. Assays were performed in duplicate. Relative fold-changes were quantified using the comparative CT (ΔΔC_T_) method [50]. Data were normalized relative to the housekeeping gene β-actin mRNA expression.

#### ***Determination of RA in cell culture media and in embryo explants by HPLC***

The media used in all experiments contained some RA i.e. from 10% FBS in DMEM and from 2% FBS in Endo-Grow, therefore it was necessary to determine their RA concentrations as well as to assess the retinoid levels in chicken embryo explants and isolated cells.

Explant and cell samples were prepared as described by Miyagi et al. [51]. Briefly, approximately 7 × 10^5^ cells were collected by trypsinization, washed with PBS and centrifuged. Pellet was resuspended and agitated for 1 min in a solution consisting of 500 μl of PBS (pH 7.4), 500 μl ethanol, 225 μl of 2 M KOH and 600 μl n-hexane. After centrifugation at 15,000 rpm for 5 min, upper phase and lower phases were collected separately. Lower (aqueous) phase (500 μl) was mixed with a solution consisting of 450 μl of 2 M HCl, 600 μl of n-hexane, centrifuged as above and the upper phase collected. Organic solvents were evaporated from collected fractions, each residue re-dissolved in 200 μl of n-hexane and subjected to HPLC analysis.

DMEM and Endo-Grow media, each containing 10 nM or 100 nM RA, were used as references for RA detection in FBS batches and in chicken embryo explants and cells. The relationship between each concentration and the height of the area under the curve of each peak was examined by linear least-squares regression analysis.

Retinoids were separated by HPLC on a reverse-phase Zorbax Eclipse XDB-C18 column 4.6 × 150 mm, 5 μm (Agilent Technologies, Germany). Column temperature was 25°C. Mobile phase was prepared by the mixing of two solutions: A (10% methanol/0.3% acetic acid) and B (90% methanol/0.3% acetic acid), in a volume ratio of A:B/1:9. Samples were injected in 100 μL. The mobile phase flow rate was 1.0 mL/min. RA was detected at 351 nm. Results were evaluated by a ChemStation Plus (Agilent, Germany). No RA was detected in the explants and in the heart forming region cells.

## Competing interests

The authors declare that they have no competing interests.

## Authors’ contributions

IC developed chicken heart forming region *in vitro* cultures, carried out all molecular studies and drafted the manuscript. UR participated in ELISA experimental design and result interpretation. MB carried out immunoassays and participated in research coordination. IN carried out all HPLC analysis. JA participated in its design discussion and helped to draft the manuscript. MHZ provided significant input in the interpretation of the data and in the revision of the manuscript. IM conceived the study, participated in its design and helped to draft the manuscript. All authors read and approved the final manuscript.

## Supplementary Material

Additional file 1 **Cakstina et al_beating cardiomyocytes.mov, 15292 K.**http://www.biomedcentral.com/imedia/2777325691142256/supp1.mov. Click here for file
